# Dysphagia aortica

**DOI:** 10.1007/s10353-021-00741-9

**Published:** 2021-11-09

**Authors:** Serena Grimaldi, Pamela Milito, Andrea Lovece, Emanuele Asti, Francesco Secchi, Luigi Bonavina

**Affiliations:** 1 Department of Biomedical Sciences for Health, Division of General and Foregut Surgery, IRCCS Policlinico San Donato, University of Milan, Piazza Edmondo Malan, San Donato Milanese, 20097 Milan, Italy; 2 Department of Radiology, IRCCS Policlinico San Donato, University of Milan, Milan, Italy

**Keywords:** Aortic dysphagia, Thoracic aortic aneurysm, Aortic pseudoanaeurysm, TEVAR, Aortoesophageal fistula

## Abstract

**Background:**

Dysphagia aortica is an umbrella term to describe swallowing obstruction from external aortic compression secondary to a dilated, tortuous, or aneurysmal aorta. We performed a systematic literature review to clarify clinical features and outcomes of patients with dysphagia aortica.

**Materials and methods:**

We searched PubMed, EMBASE, Web of Science, and the Cochrane Library. The terms “aortic dysphagia,” “dysphagia aortica,” “dysphagia AND aortic aneurysm” were matched. We also queried the prospectively updated database of our esophageal center to identify patients with aortic dysphagia referred for diagnosis and treatment over the past two decades.

**Results:**

A total of 57 studies including 69 patients diagnosed with dysphagia aortica were identified, and one patient from our center was added to the database. The mean age was 72 years (range 22–98), and the male to female ratio 1.1:1. Of these 70 patients, the majority (*n* = 63, 90%) had an aortic aneurysm, pseudoaneurysm, or dissection. Overall, 37 (53%) patients received an operative treatment (81.1% a vascular procedure, 13.5% a digestive tract procedure, 5.4% both procedures). Thoracic endovascular aortic repair (TEVAR) accounted for 60% of all vascular procedures. The postoperative mortality rate was 21.2% (*n* = 7/33). The mortality rate among patients treated conservatively was 55% (*n* = 11/20). Twenty-six (45.6%) studies were deemed at a high risk of bias.

**Conclusion:**

Dysphagia aortica is a rare clinical entity with high morbidity and mortality rates and no standardized management. Early recognition of dysphagia and a high suspicion of aortoesophageal fistula may be lifesaving in this patient population.

## Main novel aspects

- There is lack of evidence regarding definition, interpretation and management of aortic dysphagia.
- Most patients reported in the literature were diagnosed with aortic aneurysm, pseudoaneurysm, or dissection.
- Underestimation of dysphagia in this patient population may lead to death from aortoesophageal fistula.

## Introduction

Dysphagia is a common symptom reported by 10–33% of elderly individuals in the community and nursing home settings [1, 2], although the true prevalence is likely underestimated because many patients adapt through behavioral changes [3]. The most frequent causes are neurogenic, mechanical obstruction, primary motility disorder, or external compression. The term dysphagia aortica was first introduced by Pape [4] in 1932 to describe dysphagia caused by external aortic compression from an aneurysmal, dilated, or tortuous aorta [5]. In 1997, Wilkinson wrote, “The condition of dysphagia aortica is reminiscent of the Churchillian paraphrase—a riddle wrapped in a mystery inside an enigma” [6]. Dysphagia aortica is rarely mentioned in standard gastroenterological and surgical textbooks and has received little attention in the literature. Dysphagia arises when the aorta pushes the esophagus anterolaterally and against the crural diaphragm. Primary aortoesophageal fistula (AEF) is the most feared complication [7], typically in the setting of untreated thoracic aortic aneurysm (TAA) that occurs in 5–10 per 100,000 person years [8]. This may be asymptomatic and diagnosed incidentally, or it may present with symptoms due to mediastinal compression or with dissection or rupture in the worst-case scenario. Secondary AEF can occur after surgical or endovascular repair of thoracic aortic aneurysms. The typical presentation of AEF was first described by Chiari [9] as a triad of chest pain, sentinel hematemesis, and final massive hemorrhage with exsanguination after a symptom-free interval.

To date, several single case reports of aortic dysphagia have been reported, the majority in women over 70 years old with short stature, hypertension, and kyphoscoliosis [5], often in association with left ventricular enlargement and congestive heart failure [7]. The aim of the present study was to perform a literature review on dysphagia aortica, to add a case recently seen at our institution, and to highlight the diagnostic features and outcomes of this rare syndrome.

## Materials and methods

A systematic literature review was conducted to identify patients with dysphagia aortica reported from 01 January 1997 to 31 December 2020 using the PubMed, EMBASE, Web of Science, and the Cochrane Library databases. The search was conducted according to the Preferred Reporting Items for Systematic Reviews and Meta-Analysis (PRISMA) statement [10]. The following MeSH terms were used: “dysphagia AND aortic aneurysm,” “dysphagia aortica,” and “aortic dysphagia.” Two independent investigators (SG and PM) performed the literature search to identify all English-written reports. The full text of the selected studies was assessed by one investigator (SG) and classified as relevant, not relevant, or unclear. The reference lists of eligible studies were manually searched to identify additional studies. The methodological quality of the studies was assessed according to Murad et al. [11], based on a global evaluation of the most critical factors that increase the risk of bias in the specific clinical context. Disagreements at either stage were solved by discussion and arbitrated by a senior author (LB).

Data extracted included first author name, country, year of publication, number of patients included in the report, age, sex, symptoms at presentation, diagnostic methods, imaging findings, characteristics of the aneurysm, type of treatment, and short- and long-term outcomes.

The prospectively updated database of our tertiary care esophageal center was also queried to identify all patients with dysphagia as a predominant symptom referred for consultation between 2002 and 2021.

## Results

### Literature review

**Fig. 1:**
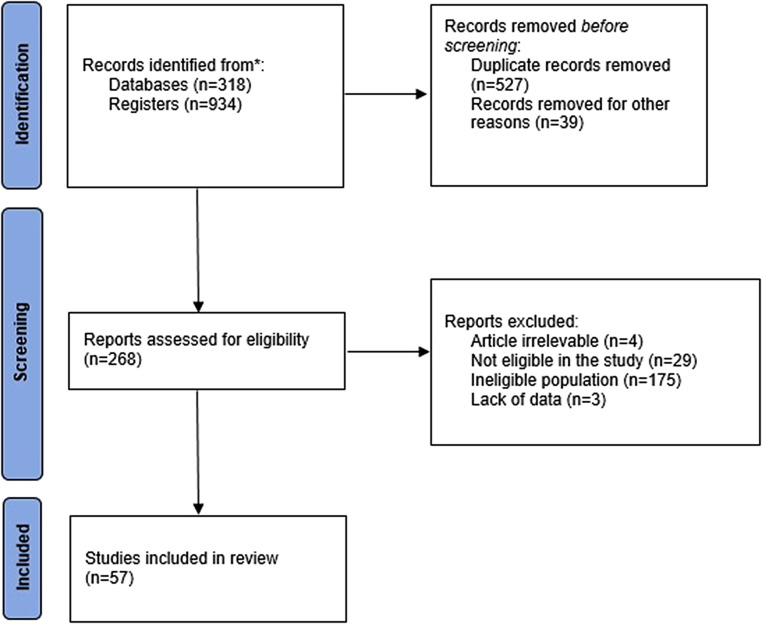
PRISMA flowchart

The search strategy identified 1252 articles (918 from registers and 318 records from databases). After duplicates were removed, 725 records were screened. Two reviewers independently screened the titles and abstracts of all papers, leading to exclusion of 984 records. A total of 57 studies were eligible for analysis (Fig. 1). There was a total of 70 patients, 33 women and 37 men, with a median age of 72 years (range 22–98). Dysphagia was associated with aortic aneurysm (*n* = 53), aortic dissection (*n* = 7), tortuous aorta (*n* = 5), or aortic pseudoaneurysm (*n* = 3). The main patient characteristics are summarized in Table 1. All patients complained of intermittent or chronic dysphagia associated with weight loss in 32.9% of cases, chest pain in 18.6%, and dyspnea in 15.7%. About half of the patients (*n* = 33, 47.1%) were considered unfit for any endoscopic or surgical approach due to elderly age and multiple comorbidities, and were mainly treated conservatively with antihypertensive therapy and a modified oral diet or through a feeding tube.

**Table 1:** Reports on dysphagia aortica from 1997 to date

First author (year)	Country	Risk of bias	Age	Sex	Characteristics of aorta	Symptoms	Treatment	Outcome/follow-up
Wilkinson JM [6] (1997)	UK	High	47	F	NR	Dysphagia	Esophageal dilation with Maloney bougies	Symptom relief/NR
			58	F	NR	Dysphagia	Dietary advice	Symptom relief/NR
Rábago G [43] (1999)	Spain	Low	62	M	TAA (MD 10 cm)	Dysphagia	Open graft repair	Symptom relief/15 months
Lau H [44] (2001)	China	Low	69	M	DTAA	Dysphagia, weight loss	NR	Death (AEF)/1 day
Taylor CW [45] (2001)	UK	Low	86	F	TAAA (MD 6 cm)	Dysphagia, weight loss	Liquid diet	Partial symptom relief/1 year
			81	F	TAAA (MD 5.5 cm)	Dysphagia, loss of appetite	Liquid diet	Partial symptom relief/2 years
Chocron S [46] (2002)	France	Low	79	M	TAA (MD 9.4 cm)	Cough, dysphagia, weight loss	TEVAR	Death (AEF)/55 days
			59	F	Aortic rupture (periaortic hematoma)	Fever, dysphagia, asthenia	TEVAR + esophagectomy	Death (AEF + sepsis)/2 months
Wedekind H [12] (2002)	Germany	High	91	F	Dissecting TAAA	Dysphagia, weight loss, dyspnea	Dietary advice and antihypertensive therapy	NR
Chiesa R [47] (2004)	Italy	Low	78	M	TAAA (MD 9 cm)	Dysphagia	Open graft repair + TEVAR	Symptom relief/1 year
Jovancević L [20] (2005)	Serbia	High	63	M	TAA	Dysphagia	NR	NR
Kutay V [21] (2005)	Turkey	High	56	M	Thoracic aortic pseudoaneurysm (6 × 8 cm)	Hemoptysis, dysphagia, chest pain	Open graft repair	NR
Contini S [48] (2006)	Italy	Low	77	F	TAA (MD 9.7 cm)	Hematemesis, dysphagia, chest pain	NR	Death (AEF)/3 days
Ebihara T [22] (2006)	Japan	High	73	M	Ruptured TAA	Cough, dysphagia	NR	NR
Petrov I [49] (2006)	Bulgaria	Low	22	F	TAA (MD 7.5 cm)	Dysphagia, voice loss	TEVAR	Symptom relief/1 year
Antón E [50] (2007)	Spain	Low	75	F	TAA (MD 4 cm)	Dysphagia, weight loss	TEVAR	Dysphagia to solids/6 months
Attaran R [23] (2007)	USA	High	56	M	TAA (MD 5.6 cm)	Dyspnea, chest pain, dysphagia	NR	NR
Hiller HG [51] (2007)	UK	Low	67	F	TAAA (MD 8.3 cm)	Dysphagia, weight loss	NR	Death (aneurysm rupture)/NR
Sebastian J [52] (2007)	India	Low	66	F	TAA	Dysphagia, weight loss, dyspnea, pneumonia	Nasogastric tube	Death (pneumonia)/4 days
Coelho-Prabhu N [34] (2009)	USA	High	87	F	TAA (MD 4 cm)	Dysphagia, weight loss	Esophageal self-expandable metal stent	Symptom relief/NR
Kim JH [5] (2009)	Korea	High	86	F	TAAA (MD 6 cm)	Nausea and vomiting, dysphagia	Liquid diet	NR
De Praetere H [53] (2010)	Belgium	Low	72	M	TAAA (MD 7.1 cm)	Thoracic pain, nausea and vomiting, dysphagia	TEVAR	Death (sepsis from esophageal necrosis)/24 days
Higuchi T [54] (2010)	Japan	Low	75	M	TAA (MD 6 cm)	Dysphagia	TEVAR	Symptom relief/3 months
Prince M [24] (2010)	Tennessee, USA	High	79	M	Dissecting TAA	Dysphagia, heartburn	Open graft repair	NR
Kische S [55] (2011)	Germany	Low	75	F	Thoracic aortic pseudoaneurysm	Dysphagia, weight loss	TEVAR	Symptom relief/2 years
Siddiqui J [56] (2011)	UK	Low	55	M	TAA (MD 7.2 cm)	Dyspnea, heartburn, dysphagia	TEVAR	Symptom relief/9 months
Cao D [57] (2012)	China	Low	69	M	Thoracic aortic pseudoaneurysm	Dysphagia, back pain	TEVAR	Symptom relief/1 month
Hori D [58] (2012)	Japan	High	68	M	TAA with “Shaggy aorta”	Dysphagia, back pain	TEVAR	Partial symptom relief/NR
Song S [59] (2012)	South Korea	Low	85	F	TAAA (MD 7 cm)	Dysphagia, chest pain, dyspnea, nausea	Soft diet and antihypertensive therapy	Symptom relief/4 weeks
Godar M [60] (2013)	China	Low	35	F	Two TAA (aortic arch and DTAA)	Dysphagia, chest pain, dyspnea	TEVAR	Mild dysphagia/2 months
Badila E [25] (2014)	Romania	High	93	F	Dissecting TAA complicated with DIC	Dysphagia, weight loss	NR	NR
Hua SR [61] (2014)	China	Low	40	F	Ruptured TAA	Dysphagia	TEVAR	Symptom relief/5 months
Skeik N [62] (2014)	USA	Low	71	M	TAA (MD 16 cm)	Dysphagia, cough	Bilateral arm compression and elevation	Death (aneurysm rupture)/1 month
Wang YP [63] (2014)	Taiwan	High	82	F	Tortuous aorta	Dysphagia, weight loss	Antihypertensive therapy	Partial symptom relief/NR
Abdul Haziz SR [64] (2015)	Brunei	High	70	F	Tortuous aorta	Dysphagia, weight loss	Soft diet and antihypertensive therapy	Intermittent transient dysphagia/NR
Al-Quthami A [65] (2015)	USA	High	29	M	Two descending thoracic aortic pseudoaneurysms	Dysphagia	Aneurysmectomy with descending thoracic interposition graft placement	Symptom relief/NR
Karavelioğlu Y [32] (2015)	Turkey	Low	98	F	TAA (MD 4.3 cm)	Dysphagia, weight loss	Soft diet and antihypertensive therapy	Symptom relief/4 weeks
Liao CY [66] (2015)	Taiwan	Low	86	M	TAA (MD 9.8 cm)	Dizziness, dysphagia, chest pain, nausea, dyspnea, acute respiratory failure	TEVAR	Death (respiratory failure, ventricular tachycardia)/2 days
Laube R [67] (2015)	Australia	Low	86	M	AAA (MD 3.7 cm)	Dysphagia, weight loss	NR	Death (aneurysm rupture)/2 days
Okamura K [68] (2015)	Japan	Low	87	M	TAA	Dysphagia, regurgitation, aspiration pneumonia	TEVAR + esophageal self-expandable covered stent	Symptom relief/1 year
Savlania A [69] (2015)	India	High	62	M	TAA	Dysphagia	Open graft repair	Symptom relief/NR
Chan YH [26] (2016)	Taiwan	High	78	F	Tortuous aorta	Dysphagia	Prokinetic agents	Death (respiratory and renal failure)/1 year
			63	F	Tortuous aorta, arthrosclerosis	Dysphagia	Soft diet	Symptom relief/NR
			72	M	Tortuous aorta, arthrosclerosis	Mild dysphagia	No treatment	NR
Ma X [70] (2016)	China	Low	22	M	Ruptured traumatic TAA	Dyspnea, dysphagia	NR	Death (aneurysm rupture)/14 days
Pitchai S [71] (2016)	India	Low	68	M	DTAA	Dysphagia, chest pain	Open graft repair	Symptom relief/6 months
			62	M	TAAA	Dysphagia, chest pain	Open graft repair	Symptom relief/6 months
			62	M	Penetrating aortic ulcer	Dysphagia	Open graft repair	Symptom relief/6 months
			40	F	DTAA (MD 6 cm)	Dysphagia	Open graft repair	Symptom relief/6 months
			59	M	DTAA	Dysphagia, chest pain	TEVAR	Symptom relief/6 months
Wang JY [27] (2016)	China	High	65	M	Dissecting TAA (MD 13.2)	Dysphagia, hoarseness	TEVAR	NR
Beqari J [72] (2017)	USA	High	82	F	TAA (MD 5.6 cm)	Chest pain, dysphagia, weight loss	Laparoscopic myotomy, division of the crus and anterior diaphragm	Symptom relief/NR
Kampitakis E [19] (2017)	Greece	High	85	F	TAA (MD 14.8 cm)	Dyspnea, dysphagia	Dietary advice	NR
Mouawad NJ [13] (2017)	USA	High	82	M	TAAA (MD 7.8 cm)	Dysphagia, weight loss, nausea	PEG	NR
Choi H [73] (2018)	Korea	High	82	M	TAA (MD 7 cm)	Dysphagia, nausea, vomiting	Liquid diet	Partial symptom relief/NR
Georgiadis GS [74] (2018)	Greece	Low	81	M	DTAA (MD 13.8 cm)	Dysphagia, weight loss, dyspnea, back pain	TEVAR	Death (pneumonia)/40 days
Gravito-Soares M [75] (2018)	Portugal	Low	78	F	TAA (MD 3.4 cm)	Dysphagia, chest pain	TEVAR	Symptom relief/6 months
Kyaw WA [76] (2018)	Brunei	Low	64	F	TAA (MD 4.6 cm)	Dysphagia, dysphonia, weight loss	No treatment	Death (septicemia from S. aureus)/4 months
Sharma M [14] (2018)	India	High	94	M	TAAA	Dysphagia, hematemesis	No treatment	Death (AEF)/2 months
			74	M	TAA (MD 5 cm)	Dysphagia, hematemesis	NR	NR
			68	M	Dissecting TAA	Dysphagia, hematemesis	Cardiothoracic surgery ns	Death (sepsis)/10 days
			54	M	Dissecting TAA	Dysphagia, hematemesis	Cardiothoracic surgery ns	Symptom relief/9 years
Choi SH [7] (2019)	Canada	Low	74	F	TAAA (MD 7.4 cm)	Dyspnea, dysphagia, retrosternal chest pain	Visceral debranching and TEVAR	Symptom relief/3 years
Elsamman MK [77] (2019)	Egypt	Low	30	M	TAA (para-aortic hematoma 5 × 6 × 10 cm)	Dysphagia	TEVAR	Symptom relief/3 days
Wang ID [15] (2019)	Taiwan	High	54	F	TAA (MD 5 cm)	Dysphagia, vomiting	NR	NR
Dejaeger M [78] (2020)	Belgium	Low	84	F	Dissecting TAA	Anorexia, weight loss, dysphagia to solids	PEG	Death (pneumonia and cardiac failure)/2 weeks
Meng Z [16] (2020)	Canada	High	89	M	TAAA (MD 6.7 cm)	Weight loss, dysphagia	Soft diet	NR
Mir AS [17] (2020)	USA	High	52	F	TAA (MD 8.3 cm)	Dysphagia, nausea and vomiting, abdominal pain	Naso-duodenal feeding tube	NR
Shrestha N [18] (2020)	Nepal	High	76	F	TAA	Dysphagia, weight loss	Liquid diet	NR
Present case (2021)	Italy	Low	80	M	TAAA (MD 6.2 cm)	Dysphagia, chest pain, weight loss	Semi-liquid diet	Death (aneurysm rupture)/4 weeks

*MD* maximum diameter, *NR* not reported, *TAA* thoracic aortic aneurysm, *TAAA* thoracoabdominal aortic aneurysm, *AAA* abdominal aortic aneurysm, *DTAA* descending thoracic aortic aneurysm, *AEF* aortoesophageal fistula, *DIC* disseminated intravascular coagulopathy, *ns* not specified

The majority (53%) of patients underwent some form of vascular, digestive tract, or combined endoscopic or surgical procedure (Table 2). A vascular procedure was performed in 30 patients and consisted of thoracic endovascular aortic repair (TEVAR) in 18, open aneurysm repair in 11, and TEVAR plus open bypass graft in 1 patient. Relief of dysphagia was noted in 20 patients (66.7%). Among the remaining patients, 5 died, 2 complained of persistent dysphagia, and 3 were lost to follow-up.

**Table 2:** Type of surgical and endoscopic procedures performed in 37 patients with dysphagia aortica

	*n*	Mortality
*Vascular procedure*	*30*	*5/27*
TEVAR	18	
Open aneurysm repair	11	
TEVAR + bypass graft	1	
*Digestive tract procedure*	*5*	*1/4*
PEG	2	
Esophageal stent	1	
Heller + crural myotomy	1	
Esophageal dilation	1	
*Combined vascular and digestive procedure*	*2*	*1/2*
TEVAR + esophageal stent	1	
TEVAR + esophagectomy	1	

*TEVAR* Thoracic Endovascular Aortic Repair

Digestive tract procedures consisted of percutaneous endoscopic gastrostomy (PEG; *n* = 2), endoscopic esophageal stent (*n* = 1), Maloney bougie dilation (*n* = 1), and laparoscopic Heller myotomy and crural myotomy (*n* = 1). The procedure was successful in 3 patients, 1 patient died, and 1 was lost to follow-up. Combined vascular and digestive procedures consisted of TEVAR and esophageal stent (*n* = 1) and TEVAR and esophagectomy. The latter was complicated by AEF and sepsis.

Follow-up data were missing for 17 (24.3%) of the patients [5, 12–27]. For the remaining 53 patients, the median follow-up was 3 months (range 2 days–9 years) and the overall mortality rate 34%. The 30-day mortality rate after TEVAR and/or open aneurysm repair was 60% (3/5). The reported reasons for death were the following: aneurysm rupture (*n* = 5), aspiration pneumonia (*n* = 5), primary AEF (*n* = 3), secondary AEF (*n* = 2), and sepsis (*n* = 3). Based on the criteria of methodological quality proposed by Murad et al. [11], 26 (45.6%) studies were considered to be at a high risk of bias.

### Case report

An 80-year-old man, body mass index (BMI) 20.1 kg/m^2^, non-smoker, was referred to our emergency department in November 2020 during the second wave of the COVID-19 pandemic. He complained of progressive dysphagia, chest pain, and 15 kg weight loss over the past 6 months. Medical history included appendectomy, prostatectomy, and prosthetic replacement of the ascending aorta via sternotomy in 2006. Laboratory tests showed hemoglobin 12.1 g/dL (normal value [n.v.] 14–18 g/dL), total protein 5.95 g/dL (n.v. 6.60–8.70 g/dL), albumin 3.1 g/dL (n.v. 3.50–5.20 g/dL), C‑reactive protein 12.6 mg/dL (n.v. < 0.5 mg/dL). A transthoracic echocardiogram showed dilatation and systolic dysfunction of the left ventricle (ejection fraction 33%), and mild aortic insufficiency.

**Fig. 2:**
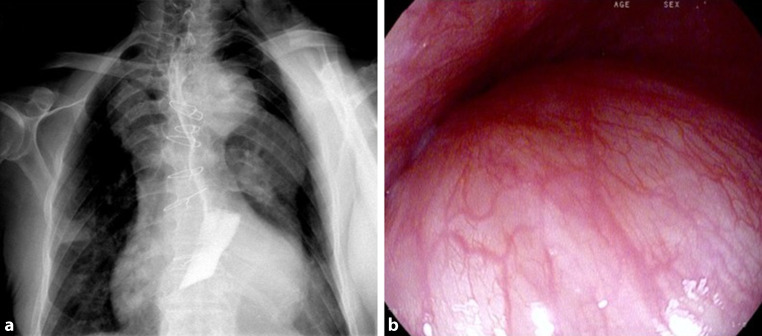
**a** Barium swallow study showing a thin transit of contrast due to aneurysm compression over the distal esophagus. **b** Upper gastrointestinal endoscopy showing pulsatile bulging from aortic compression

**Fig. 3:**
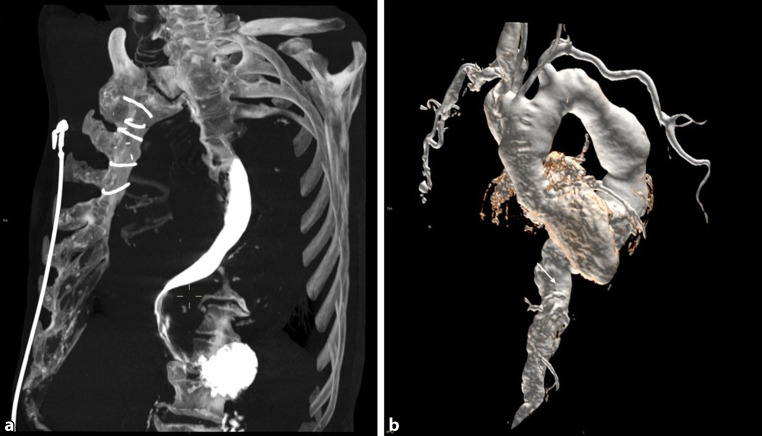
**a** CT scan after oral contrast administration with maximum intensity projection reconstruction showing esophageal compression. **b** Magnetic resonance angiography after oral administration of gadolinium-based contrast, showing the whole anatomy of the aortic aneurysm lumen with a virtual rendering technique

A barium swallow study revealed a marked extrinsic compression at the level of the lower third of the esophagus, with a filiform contrast flow and dilatation above. Esophagogastroduodenoscopy confirmed a pulsatile extrinsic compression with luminal narrowing from 38 cm to 42 cm from the dental arch (Fig. 2). A computer tomography (CT) scan performed with oral contrast medium showed distal esophageal compression due to a giant thoracic aortic aneurysm (Fig. 3a). Magnetic resonance angiography (MRA) confirmed a giant aneurysm extending from the ascending aorta to the infrarenal region, with signs of intravascular thrombosis and perivascular reaction. The diameter of the aorta was 51 × 57 mm in the ascending thoracic portion, 48 × 46 mm at the aortic arch, 57 × 62 mm in the mid-third of the descending aorta, and 36 × 35 mm below the level of the renal arteries (Fig. 3b).

**Fig. 4:**
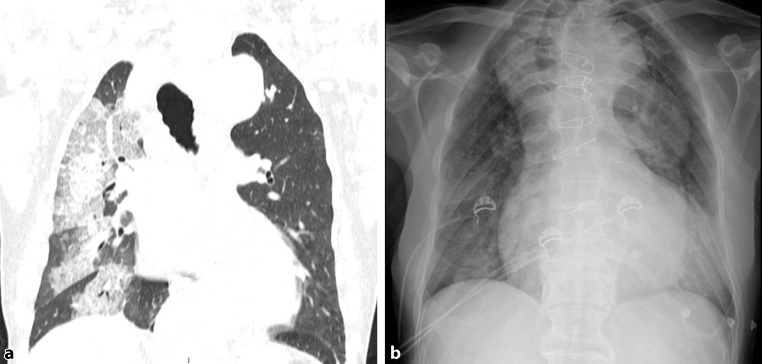
**a** Thoracic CT scan showing right pulmonary consolidations as a sign of aspiration pneumonia. **b** Chest X‑ray showing resolution of pneumonia

Based on the above findings, further diagnostic work-up with high-resolution esophageal manometry was considered to exclude concomitant achalasia. However, on the second day of the hospital stay, the patient acutely complained of dyspnea at rest with 90% of SpO_2_ in ambient air. Oxygen therapy was started at 2 L/min. Arterial blood gas analysis showed pH = 7.43, pCO_2_ = 36.3 mm Hg, pO_2_ = 59.5 mm Hg, HCO_3_ = 24 mmol/L, and sO_2_ = 89.1%. Laboratory tests for *Legionella pneumophila, Streptococcus pneumoniae*, and SARS-CoV‑2 RNA swab and IgG and IgM were negative. A chest CT scan revealed signs of right lung aspiration pneumonia. Antibiotic therapy was started (piperacillin/tazobactam 4.5 g four times a day) and oxygen therapy was increased (Venturi mask 35%, 8 L/min). Due to the increasing need of oxygen therapy, the patient was switched to continuous positive airway pressure (CPAP) therapy with significant improvement of SpO2. Oxygen flow was then progressively reduced and the chest X‑ray after 2 weeks of antibiotic therapy revealed complete resolution of the clinical and radiologic pattern (Fig. 4).

The conclusion of a multidisciplinary board meeting including general and vascular surgeons was that the patient was unsuitable for vascular repair given his age, the multiple comorbidities, the low ejection fraction, and the aortic anatomy. The patient refused to undergo esophageal manometry. Therefore, enteral nutrition through a nasogastric tube or percutaneous endoscopic gastrostomy was recommended, but the patient declined any invasive procedure. He was then discharged on a semi-liquid diet. The patient died at home 4 weeks after hospital discharge due to probable aneurysm rupture.

## Discussion

In the present systematic review, dysphagia aortica was associated with thoracic aortic aneurysm in most patients. Interestingly, 21 of 63 (33.3%) patients underwent TEVAR as a single treatment modality or combined with other vascular or digestive tract procedures.

The prevalence of dysphagia aortica is neither well reported nor well studied [7]. It has been suggested that external compression of the esophagus may not represent the major pathophysiological mechanism, but rather an incidental finding. As in dysphagia lusoria, an underlying esophageal motility disorder may be present in some of these patients, particularly in those without evidence of aneurysm [28, 29]. It has also been speculated that long-lasting esophageal compression may evolve into esophageal pseudoachalasia, a rare condition accounting for less than 5% of patients with achalasia-like syndrome [30, 72, 78]. Reported findings at esophageal manometry are low-amplitude propagated peristaltic waves in the proximal esophagus and a localized high-pressure zone at the site of vascular compression. Wilkinson [6] investigated 5 patients complaining of dysphagia to solids associated with a localized high-pressure zone on esophageal manometry. None of the patients had an aneurysm, and videoradiographic assessment with a solid bolus supported the diagnosis of dysphagia aortica.

Considering the rarity of dysphagia aortica, there is no gold standard for diagnosis and therapy. A history of aortic aneurysm or prior aortic graft or TEVAR is key for diagnosis. Radiological and endoscopic imaging provides a high index of suspicion [5]. The diagnostic work-up should include chest X‑ray, upper gastrointestinal endoscopy, barium or videofluoroscopic swallowing study, chest CT scan with oral and intravenous contrast, and esophageal manometry. No single diagnostic tool can definitively prove the diagnosis of dysphagia aortica. Radiographic findings may be inconclusive because a dilated and tortuous aorta is frequently seen in elderly patients in the absence of a true aneurysm. Upper gastrointestinal endoscopy has the potential to exclude other possible causes of upper gastrointestinal bleeding, and to detect signs of AEF such as small mucosal erosions, oozing from a pin-hole erosion, ulcer with adherent clot over a pulsatile mass, or graft exposure [31].

**Fig. 5:**
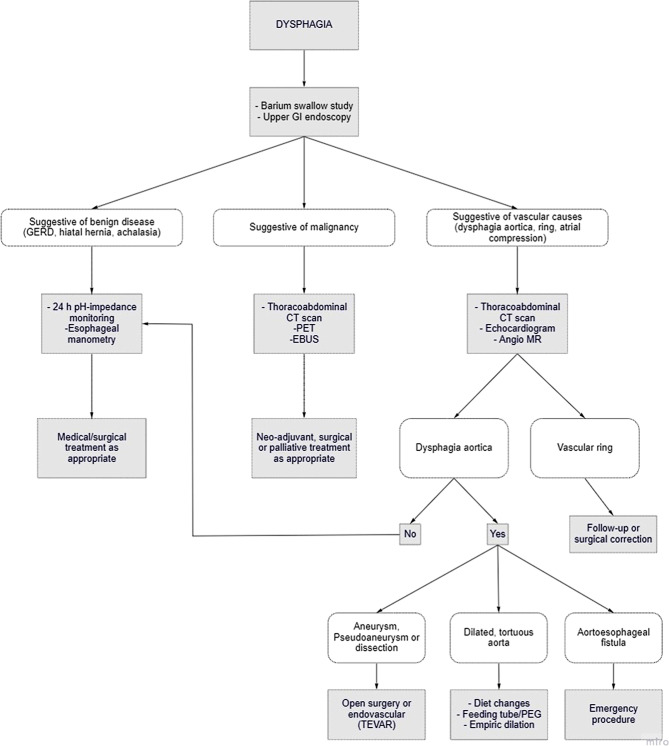
Proposed management algorithm for patients with aortic dysphagia. *GERD* Gastroesophageal reflux disease, *TEVAR* Thoracic Endovascular Aortic Repair, *PEG* Percutaneous Endoscopic Gastrostomy

The treatment of dysphagia aortica depends on the severity of symptoms and the patient’s comorbidities. Most patients with mild and intermittent symptoms may be treated conservatively by a modified diet. Treatment of associated cardiac failure or arterial hypertension may also significantly reduce the burden of symptoms, especially in the case of small aneurysms [6, 32]. Percutaneous endoscopic gastrostomy, endoscopic esophageal dilation with bougie [6, 33], or an esophageal self-expandable stent have been used sporadically [34]. In the past, surgical procedures proposed to reduce esophageal compression included anterolateral transposition of the esophagus with posterior cruroplasty [33], and mobilization of the distal esophagus from the aortoesophageal decussation area with creation of a posterior pleural sling [35]. More recently, Heller myotomy with division of the right crus of the diaphragm to relieve esophageal compression [33–36] has also been reported. A proposed management algorithm for dysphagia aortica is shown in Fig. 5.

Aortic aneurysm is a progressive disease, and current practice guidelines recommend treatment of asymptomatic thoracic aortic aneurysms with diameter ≥ 55 mm. Aortoesophageal fistula is a rare complication, representing less than 10% of all aortoenteric fistulas [37]. In 1991, Hollander and Quick [38] reviewed 500 patients with AEF and found that an untreated thoracic aortic aneurysm accounted for 54% of the cases. Since then, secondary AEF have become an increasingly recognized complication of surgical or endovascular repair of thoracic aortic aneurysms.

Direct open surgical aneurysm repair and TEVAR [7, 39] are the most common therapeutic options in patients who are fit for these procedures. Over the past 20 years, TEVAR has evolved into an upfront treatment option, showing the potential for preventing further aortic enlargement and ultimate aortic rupture. However, the fact that periaortic hematoma remains untreated may potentially aggravate dysphagia. In addition, further impingement of the esophagus by the hematoma or by an oversized stent, endoleak, stent migration, or a penetrating aortic ulcer can accelerate aortic rupture, AEF, and sepsis [40, 41].

In a large multicenter survey [42] including 1138 patients treated with TEVAR over a 10-year period, 2 of 25 (8%) patients with aortoesophageal or aortobronchial fistula presented with dysphagia. The interval between the first reported episode of sentinel hemorrhage and the final diagnosis ranged from 2 h to 6 months. Thirty-day mortality and actuarial 2‑year survival were 28% and 54.7%, respectively. A combined endovascular and surgical approach reduced infectious complications and recurrent fatal bleeding. Although the evidence was not strong enough to justify changes in clinical practice, the authors felt that this complication was underestimated even in large trials and questioned the utility of the endovascular approach as the exclusive therapeutic modality.

Since the occurrence of secondary AEF complicating TEVAR is unpredictable, it would be paramount to establish the criteria for an early diagnosis. Unfortunately, the association of dysphagia with thoracic aortic aneurysm remains elusive in most reported series, often because the symptom is mild, intermittent, or neglected by both the patient and the physician. Further studies are needed to establish the prevalence of subclinical dysphagia aortica by using specific symptom questionnaires before and after aneurysm repair. Moreover, dysphagia should be rightfully included in the Chiari’s triad that originally reported chest pain as the initial manifestation of AEF [9], as well as in clinical practice guidelines [40, 79]. Interestingly, the interval between the onset of dysphagia and bleeding from AEF exceeded 1 month in the few reported patients [48]. It is possible that with increasing worldwide adoption of the endovascular procedures, the reported incidence of dysphagia and AEF may increase as well [80]. This may temper the enthusiasm for TEVAR, which should instead represent a bridge to definitive aortic and esophageal reconstruction in patients who are fit for a staged procedure.

This review has several limitations, including reporting bias and the fact that all studies were case reports including up to 5 patients. Therefore, a significant gap in clinical evidence for both diagnostic and therapeutic outcomes remains due to the heterogeneity and the average low methodological quality of the case reports.

## Conclusion

Dysphagia aortica is a rare entity with a high mortality rate and no standardized management. Lack of awareness and symptom underestimation may contribute to diagnostic delay. A thorough investigation is recommended to exclude other causes of dysphagia. With modern diagnostic technologies, dysphagia aortica should no longer represent an clinical enigma. One- or two-stage aneurysm repair is feasible in selected patients and may prevent AEF. Surveillance of patients with thoracic aortic aneurysms, early recognition of dysphagia, and a high suspicion of AEF may be lifesaving.
